# Haliscosamine: a new antifungal sphingosine derivative from the Moroccan marine sponge *Haliclona viscosa*

**DOI:** 10.1186/2193-1801-2-252

**Published:** 2013-06-04

**Authors:** Belkassem El-Amraoui, Jean-Fançois Biard, Aziz Fassouane

**Affiliations:** Faculty of Science, University of Chouaib Doukkali, El-Jadida, Morocco; MMS Research Group, Faculty of Pharmacy, University of Nantes, Nantes, France; Director of National School of Business and Management (ENCG), El Jadida, Morocco

**Keywords:** Haliscosamine, *Haliclona*, *Candida*, *Cryptococcus*

## Abstract

In the aim of searching for new antifungal products from marine origin, we have isolated a sphingosine derivative, (*9Z*)-2-amino-docos-9-ene-1,3,13,14-tetraol (Haliscosamine) from the Moroccan sea sponge *Haliclona viscosa* using bio-guided (antifungal) HPLC methods. The molecular structure of this compound was elucidated by spectrometric techniques IR, UV, MS and NMR. The isolated metabolite showed a significant antifungal activity against *Cryptococcus* and *Candida* species and a weak general toxicity in the brine shrimp lethality test. Further research is needed to study its *in vivo* activity, as well as to elucidate the mechanism underlying its activity in the hope of a future use in medical mycology.

## Introduction

Our laboratory aims to find new antifungal metabolites from marine origin for use in human medicine on one hand, and on the other hand in phytopathology. Marine invertebrates of Moroccan Atlantic coast are our preferred source of producers of active substances, specifically sponges, known generally to contain secondary metabolites with interesting biological activities (Faulkner 2002) including antimicrobial (Baker et al. 2009), antifungal (Clark et al. 2001), antileishmanial (Dube et al. 2007), antioxidant (Regoli et al. 2004) and cytotoxic activities (Ayyad et al. 2009 Fusetani et al. 1989 Erickson et al. 1997 Rashid et al. 2000). In the first work (El-Amraoui et al. 2010), we screened antifungal activity in hydroalcoholic and organic extracts of 14 sponges and showed that three species of them are active against pathogenic fungi and bacteria: *Haplosclerida adocia, Cinachyrella tarentina* and *Haliclona viscosa.* This latter species being shown the most active, we chose it to isolate the active compound. Kupchan partitioning, then multistep HPLC from the organic extract provided a pure active product. We determined its structure and evaluated its antifungal potential and its toxicity.

## Results

### Isolation of the active product

Lyophilized sponge (800 g) was extracted with EtOH, the extract partitioned between CH_2_Cl_2_ and H_2_O, and the organic solution submitted to a Kupchan liquid partition procedure. EtOAc and MeOH:H_2_O fractions were pooled, then the mixture was successively separated by three steps of HPLC to yield 47 mg of an amorphous pale yellow product. All steps of this isolation were bio-guided by antifungal (agar disc-diffusion) test. The total mass of the product (taking into account other fractions containing isolated compound) was estimated to be 80 mg from 800 g of dry sponge (0.01%).

### Molecular structure of the product isolated from *Haliclona viscosa*

**Figure 1:**
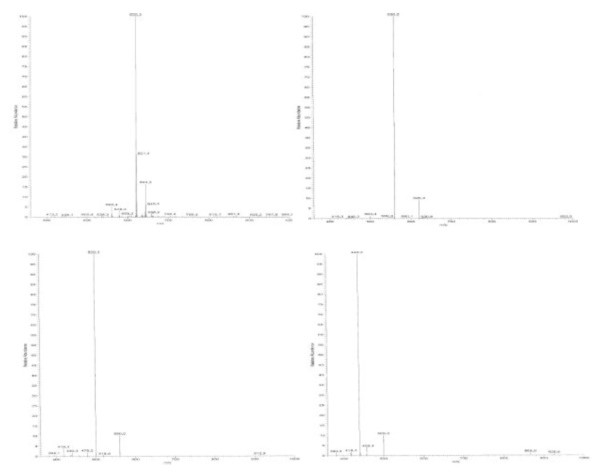
**Masse spectrum of acetylated compound.**

The molecular formula of the compound was determined to be C_22_H_45_NO_4_, indicating one degree of unsaturation, by HRESIMS with the molecular ion peaks at *m/z* 410.3255 ([M + Na]^+^) for C_22_H_45_NO_4_Na (calculated 410.32463, δ 2ppm), and *m/z* 388.3431 ([M + H]^+^) for C_22_H_46_NO_4_ (calculated 388.34268, δ 1ppm) in positive ion mode. MS analysis of the product ([M + H]^+^, 388) showed fragments at 370, 352, 334, 316 for the loss of four –OH, and 299 for the –NH_2_. Same analysis was done with the acetylated product showing a [M + Na]^+^ ion at *m/z* 620 consistent with a penta-acetylated product, and fragments at 560, 500, 440 and 380 for the losses of four acetyl moieties (Figure 1). No ion at 320 for a fifth acetyl was observed.

**Figure 2:**
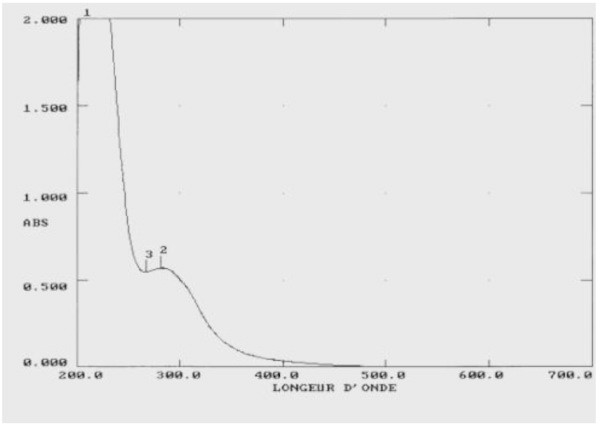
**UV-spectrum of haliscosamine.**

The UV spectrum (in MeOH) (Figure 2) showed weak absorptions (δ = 1178 at 207.5 nm and δ = 228 at 281.0 nm).

**Figure 3:**
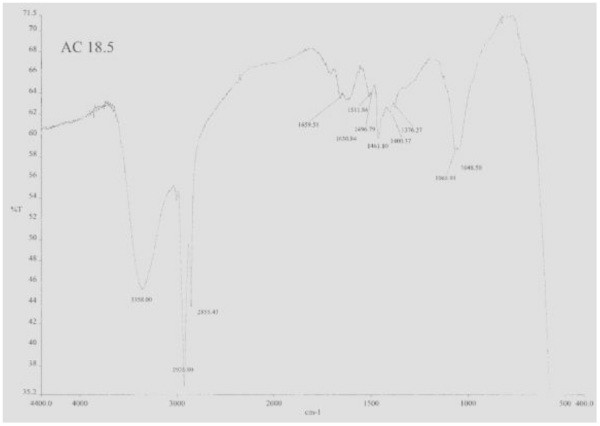
**IR spectrum of haliscosamine.**

The IR spectrum (Figure 3) showed absorptions at 3358, 1065 and 1048 (hydroxyl), 2926 and 2855 (aliphatic), 1659 and 1630 cm^-1^ (double bond). No peak was visible for a carbonyl function.

**Figure 4:**
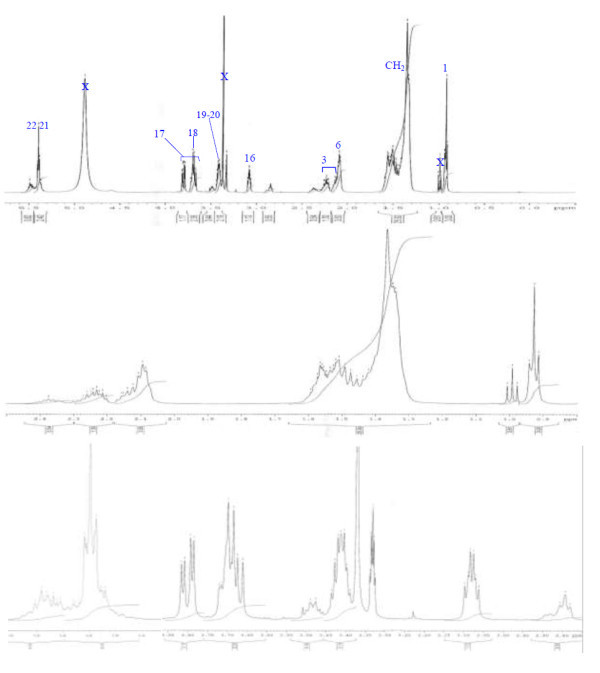
^**1**^**H NMR spectrum of haliscosamine.**

**Figure 5:**
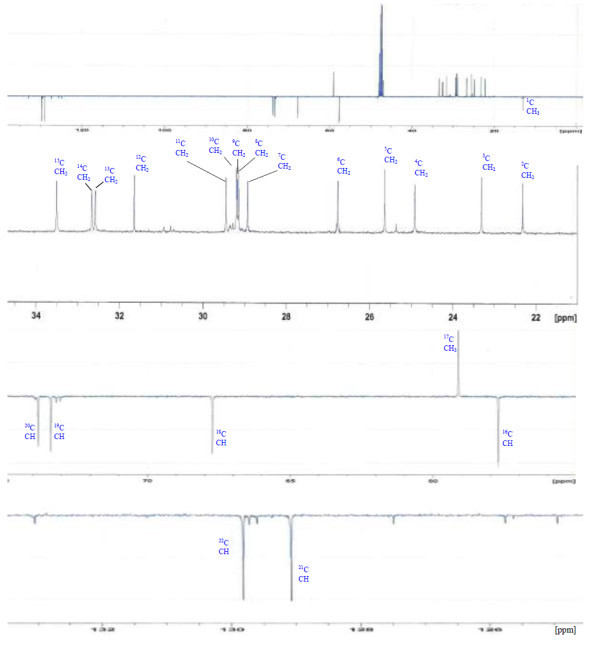
^**13**^**C NMR spectrum of haliscosamine.**

**Figure 6:**
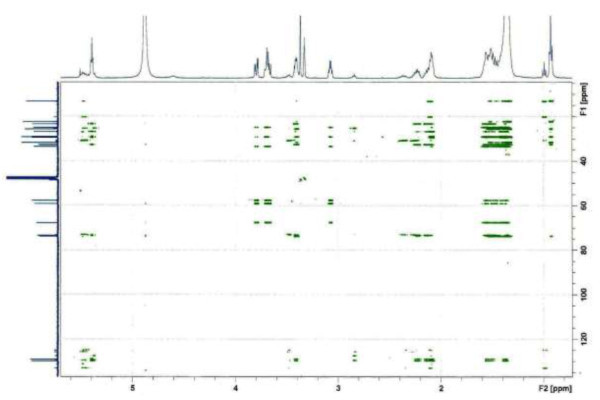
**HSQC spectrum of haliscosamine.**

**Figure 7:**
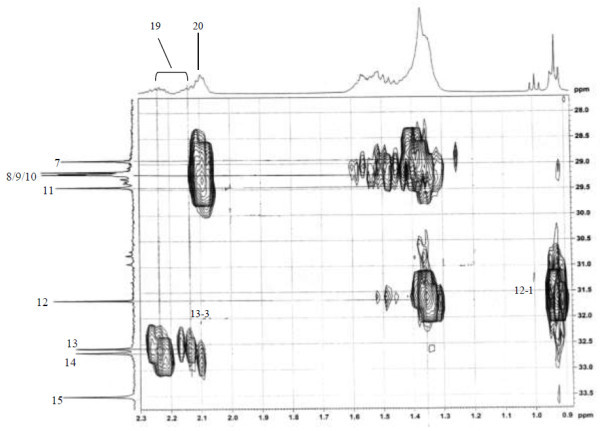
^**1**^**H-**^**13**^**C HMBC spectrum of haliscosamine (MEOD).**

**Figure 8:**
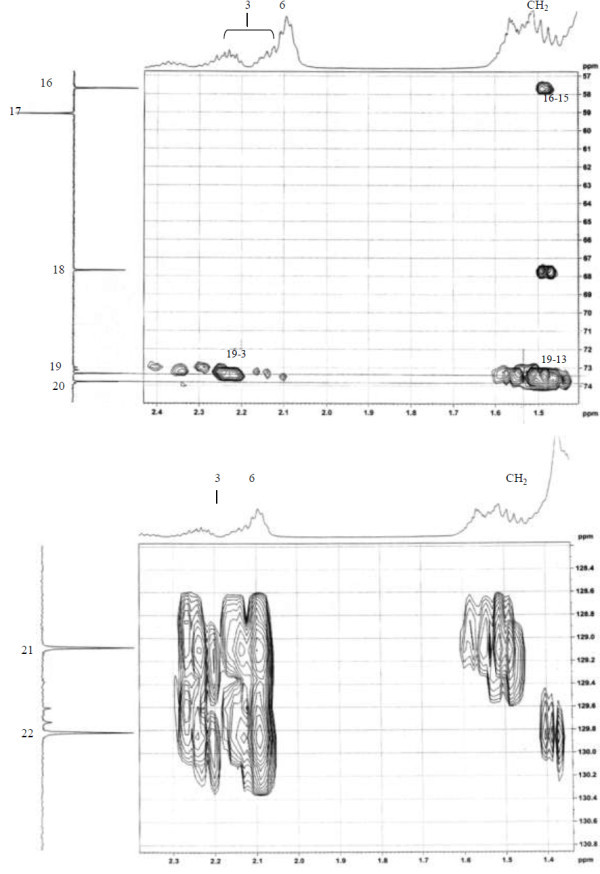
^**1**^**H-**^**13**^**C HMBC spectrum of haliscosamine (MEOD).**

**Figure 9:**
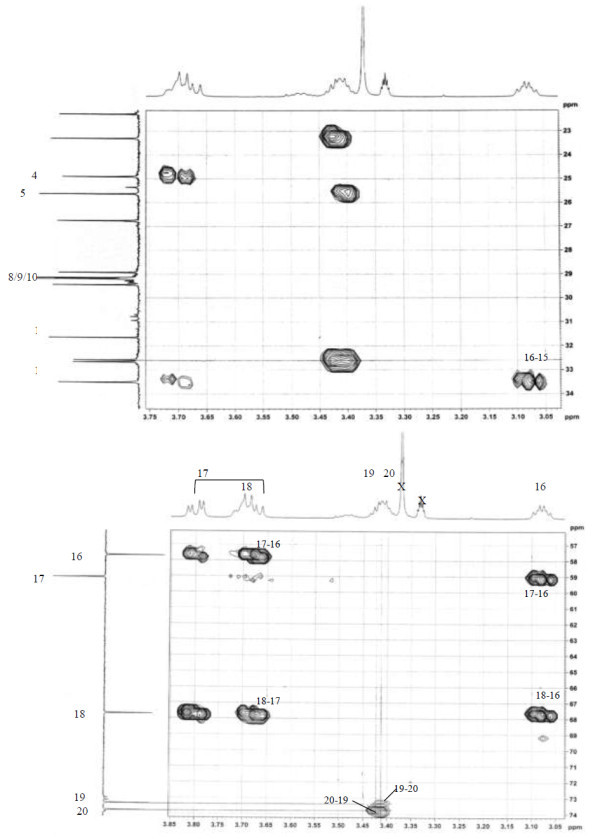
^**1**^**H-**^**13**^**C HMBC spectrum of haliscosamine (MEOD).**

**Figure 10:**
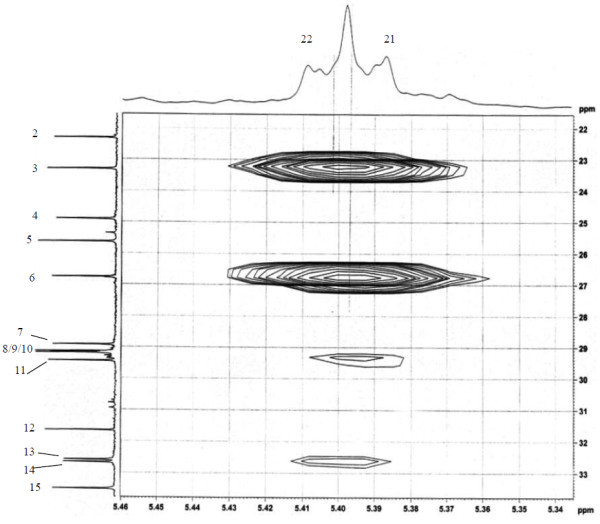
^**1**^**H-**^**13**^**C HMBC spectrum of haliscosamine (MEOD).**

**Figure 11:**
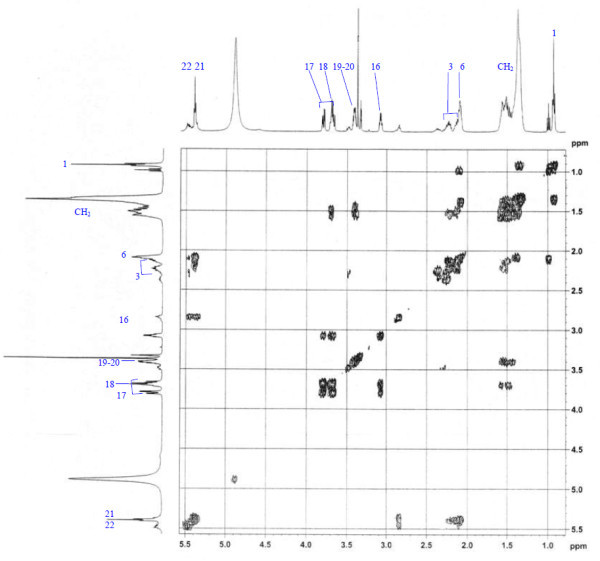
^**1**^**H-**^**1**^**H COSY spectrum of haliscosamine.**

**Figure 12:**
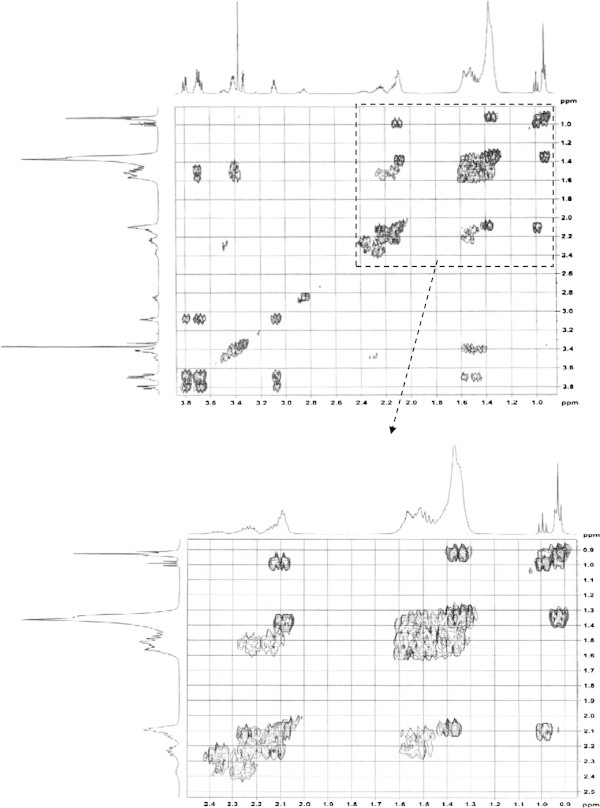
**Detail of**^**1**^**H-**^**1**^**H COSY spectrum of haliscosamine.**

**Figure 13:**
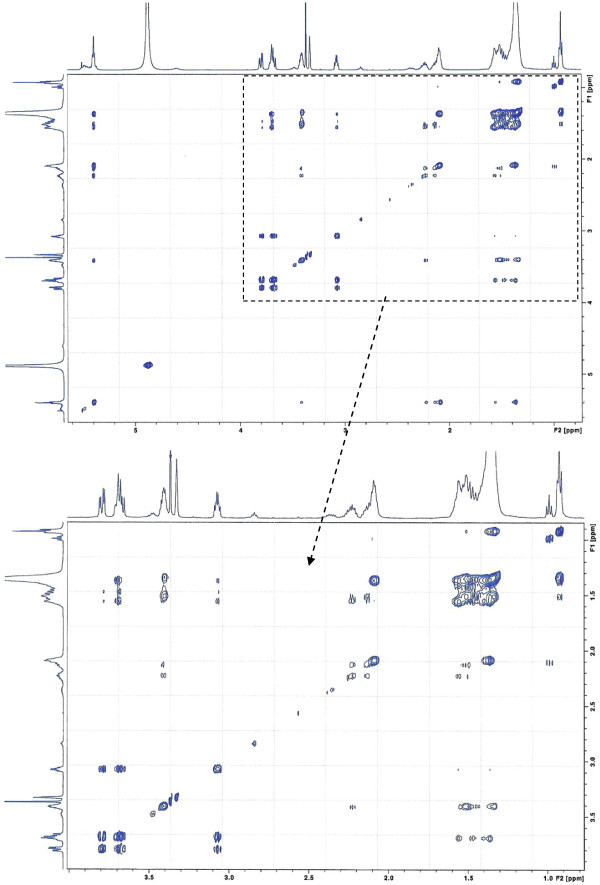
**TOCSY spectrum of haliscosamine.**

**Figure 14:**
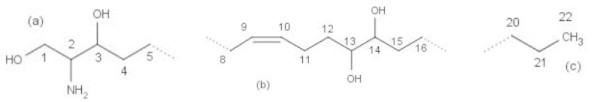
**The three sub-structures of haliscosamine.(a)** C-1 to C-6, **(b)** C-8 and C-16 **(c)** C-20 to C-22.

**Figure 15:**
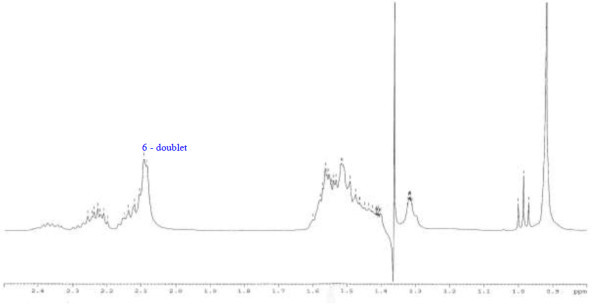
^**1**^**H spectrum of haliscosamine (MEOD, irradiation at δ 1.369 ppm).**

**Figure 16:**
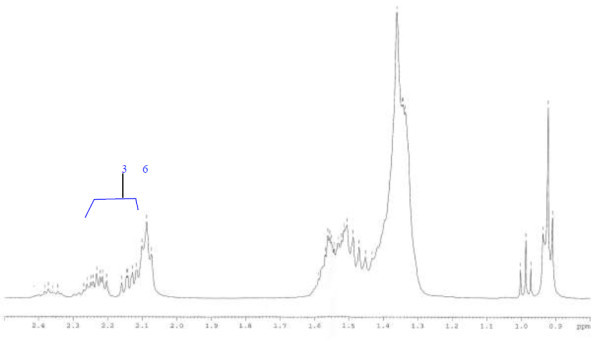
^**1**^**H spectrum of haliscosamine (MEOD, irradiation at δ 5.398 ppm).**

**Figure 17:**
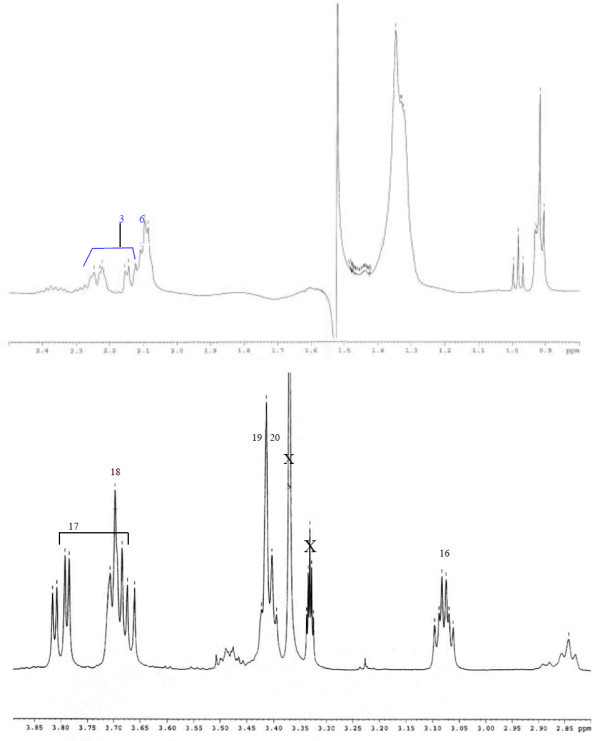
^**1**^**H spectrum of haliscosamine (MEOD, irradiation at δ 1.523 ppm).**

**Figure 18:**
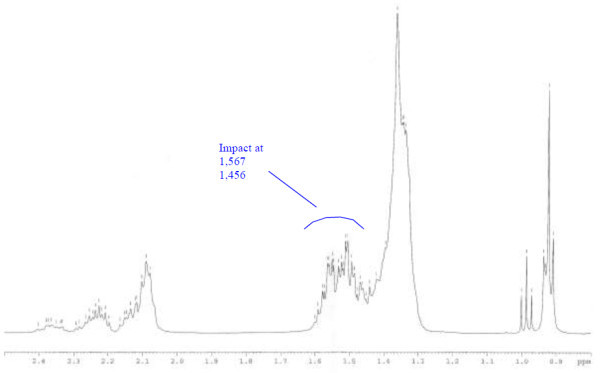
^**1**^**H spectrum of haliscosamine (MEOD, irradiation at δ 3.415 ppm)**

Analysis of ^1^H (Figure 4), ^13^C (Figure 5) and HSQC (in deuterated methanol CD_3_OD) (Figure 6) experiments revealed the presence of 22 carbons with 39 protons, including one methyl, 15 methylens and six methines. Table 1 shows NMR spectroscopic data of compound. The presence of four oxygenated C atoms at 59.11 (C-1), 67.72 (C-3), 73.36 (C-13), 73.81 (C-14), a probably nitrogenated C atom at 57.72 (C-2), and two double bonded carbons at 129.08 (C-9) and 129.82 (C-10) were elucidated. General appearance of the NMR spectra suggested that it is a derivative of the sphingosine. Therefore, the six remaining protons should correspond to one –NH_2_ and four –OH. From the careful examination of the 2D NMR experiments spectra (HMQC, HMBC (Figures 7, 8, 9 and 10), COSY (Figures 11 and 12), TOCSY (Figure 13)), three different structural units were identified: C-1 to C-5, C-8 to C-16 and C-20 to C-22 (Figure 14). However, respective positions of the carbons 9/10, 8/11 and 13/14 were not clear because there was a strong overlapping of their signals on the ^1^H-NMR spectrum. Experiments with other solvents were limited by the poor solubility of compound in AcN, CH_2_Cl_2_ and CHCl_3_. Finally, the assignments of the ^1^H-NMR signals were determined by recording a new set of experiments with increased resolution for the corresponding areas, and by extensive ^1^H-NMR decoupling experiments (Figures 15, 16, 17 and 18). Consequently, clear relationships between all carbons were established for these three partial structures.

**Table 1:** **NMR spectroscopic data for haliscosamine**

Carbon	δ^13^C ppm	δ ^1^H ppm ***J(Hz)***		HMBC	COSY	TOCSY
1a	59.12, CH2	3.67dd	(*J = 11.7, 4.0*)	2, 3	1b, 2, 3	2, 3
1b		3.79dd	(*J = 11.7, 4.0*)	2, 3	1a, 2, 3	2, 3
2	57.72, CH	3.07 m	(*J = 6.8, 4.0*)	1, 3, 4	1	1, 3
3	67.72, CH	3.69 m	(*J = 6.8, 4.0*)	1, 2, 4, 5	1, 4	1, 2, 4, 6
4a	33.51, CH2	1.47 m		2, 3, 5		
4b		1.57 m		2, 3, 5		
5a	24.92, CH2	1.40 m		3		
5b		1.57 m		3		
6	28.94, CH2	1.37 m				
7	29.45, CH2	1.37 m		8	8	9
8	26.77, CH2	2.09 m		7, 9	7, 9, 10	
9	129.08, CH	5.39 m		8	8, 10	8, 7
10	129.82, CH	5.40 m		11	8, 9, 11	11, 13
11a	23.32, CH2	2.13 m		10, 12, 13	10, 12	10, 13
11b		2.24 m		10, 12, 13	10, 12	10, 13
12a	32.59, CH2	1.54 m		11, 13		13
12b		1.59 m		11, 13		13
13	73.37, CH	3.42		11, 12, 14, 15		10, 11, 12, 15
14	73.81, CH	3.40		13, 15, 16	15	
15a	32.67, CH2	1.44 m		13, 14, 16	14	13
15b		1.54 m		13, 14, 16	14	13
16a	25.64, CH2	1.37 m		14, 15		
16b		1.52 m		14, 15		
17	29.17, CH2	1.37 m				
18	29.18, CH2	1.37 m				
19	29.20, CH2	1.37 m				
20	31.66, CH2	1.33 m		21, 22		
21	22.32, CH2	1.36 t	(*J = 6.5*)	20, 22		
22	13.06, CH3	0.92		20, 21		

**Figure 19:**
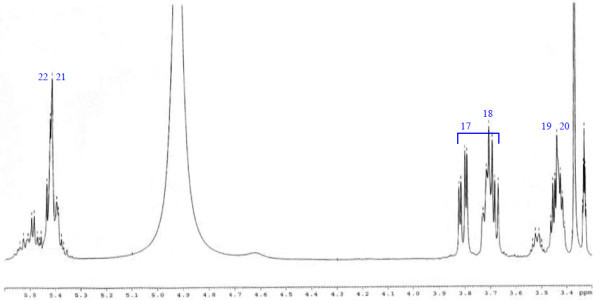
^**1**^**H spectrum of haliscosamine (MEOD, irradiation at δ 2.09).**

**Figure 20:**
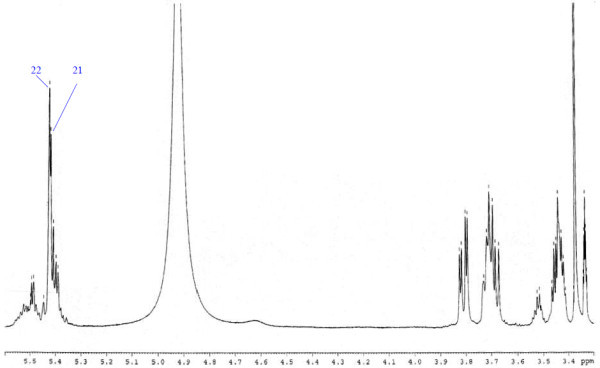
^**1**^**H spectrum of haliscosamine (MEOD, irradiation at δ 2.28).**

**Figure 21:**
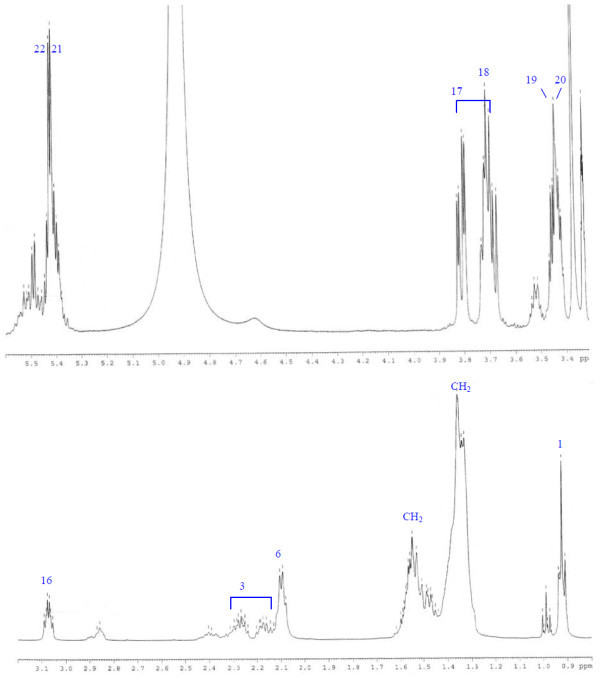
^**1**^**H NMR spectrum of haliscosamine (CD3OD + few drops of C6D6).**

Assemblage of the three sub-structures was done by supplementary ^1^H-NMR decoupling experiments, which allow to link C-22 to C-21 and C-20, then to C-16 by the way of the three CH_2_ at 29.17, 29.18 and 29.20 (10, 9, 8 respectively). Accordingly, the two last remaining –CH_2_ (C-6 and C-7) should be positioned between C-5 and C-8 to complete the structural elucidation of compound. Geometry of the double bond was assigned to be Z on the basis of the ^1^H-NMR experiments: Irradiations at δ2.09 (Figure 19) (H’s of C-8) and 2.28 (Figure 20) (H’s of C-11) resulted in a change of the olefinic protons group and allowed to found a 10 Hz coupling constant for these protons. Recording of other ^1^H-NMR spectrum in CD_3_OD with some drops of deuterated benzene (C_6_D_6_) (Figure 21) increases the resolution and allowed confirming this 10 Hz coupling constant. The geometry of the double bond was confirmed by the weak difference between the chemical shifts values of the carbons C-8 (26.77) and C-11 (23.32).

The optical rotation was [α]^20^_D_ = 22,3 (c = 0,76, MeOH).

**Figure 22:**
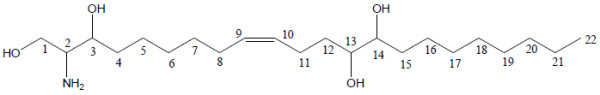
**Molecular structure of haliscosamine [(9Z)-2-amino-docos-9-ene-1,3,13,14-tetraol].**

The compound isolated from *H. viscosa:* (*9Z*)-2-amino-docos-9-ene-1,3,13,14-tetraol (Figure 22), is a new sphingosine derivative. Therefore, we named this product haliscosamine.

### Biological activities of haliscosamine

#### Antifungal activity

*In vitro* antifungal activity and minimal inhibitory concentration (MIC_90_) of haliscosamine against the pathogenic yeasts were reported in Tables 2 and 3 respectively.

**Table 2:** ***In vitro*antifungal activity of haliscosamine and nystatin against pathogenic yeasts**

Test organism	Growth inhibition diameter (mm)		
	Haliscosamine (100 μg)	Nystatin (100 μg)	(-)-untenospongin B (100 μg)
*C. neoformans*	28	24	0
*C. albicans*	25	25	17
*C. tropicalis*	25	0	5

**Table 3:** **Minimum Inhibitory Concentration (MIC**_**90**_**) of haliscosamine and nystatin against pathogenic yeasts**

Test organism	Minimum inhibitory concentration (μg/mL)	
	Haliscosamine	Nystatin
*C. neoformens*	0.2 – 0.4	3.12 – 6.25
*C. albicans*	0.4 – 0.8	3.12 – 6.25
*C. tropicalis*	0.4 – 0.8	0

The *in vitro* antifungal activity by the diffusion method showed that haliscosamine was more active than nystatin in inhibiting the growth of C*ryptococcus neoformans* (ATCC 11576), but showed the same activity as nystatin in inhibiting the growth of *C. albicans* (ATCC 10231). On the other hand, haliscosamine was active against *C. tropicalis* (R2 CIP 1275.81), an amphotericin B and nystatin resistant strain.

The MIC_90_ of haliscosamine is less than that of nystatin on *C. neoformans*. and *C. albicans*, and the same level as the previous in *C. tropicalis*.

#### Toxicity test

Lethality concentration (24 h-LC_50_ value) for haliscosamine is 664.86 μg/mL. This general toxicity activity is considered weak when the LC_50_ was between 500 and 1000 μg/mL, moderate when the LC_50_ was between 100 and 500 μg/mL, and designated as strong when the LC_50_ ranged from 0 to 100 μg/mL (Padmaja et al. 2002 Canales et al. 2007). Consequentely, the toxicity of the haliscosamine may be considered as weak by this test.

## Discussion

Haliscosamine isolated from the Moroccan marine sponge *Haliclona viscosa* is a new derivative of sphingosine with an original molecular structure ((Z)-2-amino-docos-9-ene-1,3,13,14-tetraol, C_22_H_45_NO_4_). However, the relative and absolute stereochemistries of the molecule remain to be determined, with confirmation by total synthesis.

The high content (0.01%) of the haliscosamine in the sponge, as its presence in the free state and not involved in a ceramide or other complex lipid is remarkable. The haliscosamine is added to products already isolated from sponges of the genus *Haliclona*, such the alkaloids viscosamine (Volk and Kock 2003), viscosaline (Volk and Kock 2004) and haliclamines A, B, C and D (Fusetani et al. 1989 Volk et al. 2004). The activity of this product also shows the bioactive potential of marine sponges from the Atlantic Coast of Morocco, and encourages them to continue our sorting activity. The only study that has been done before has been the isolation of untenospongin B (antimicrobial) from the marine sponge *Hippospongia communis* (Rifai et al. 2004) and of fasciculatin (cytotoxic and inhibitor of lymphocyte proliferation) isolated from *Ircinia variabilis* (Rifai et al. 2005).

Haliscosamine has a remarkable antifungal effect. Sphingolipids are already known for their antiseptic and antifungal activity (Bibel et al. 1995), and sphingosine and its derivatives are natural antimicrobial agents, protecting the human skin from bacterial colonization (Bibel et al. 1993) as well as being anti-inflammatory agents (Radhika et al. 2005).

Compared to nystatin, haliscosamine showed *in vitro* significant activity against *Candida albicans* (ATCC 10231), *Candida tropicalis* (CIP 1275.81) and *Cryptococcus neoformans* (ATCC 11576). These three yeasts are often involved in human mycology especially *C. tropicalis* that is resistant to nystatin and amphotericin B.

Comparing the antifungal activity of haliscosamine with (-)-untenospongin B isolated from the marine sponge *Hippospongia communis* collected from the Atlantic Coast of Morocco (Rifai et al. 2004), it was found that haliscosamine was more active than untenospongin B in inhibiting the growth of *C. tropicalis* (R2 CIP 1275.81), *C. albicans* (ATCC 10231) and *C. neoformans* (ATCC 11576) (Table 2). So, haliscosamine is an interesting product, thanks to its antifungal potential against strains involved in human mycology, including those resistant to nystatin. Further studies are still needed to determine *in vivo* activity and toxicity of haliscosamine in the animal model as well as to elucidate the mechanism underlying its activity. The lethal concentration (24 h-LC_50_ = 664.86 μg/mL) of haliscosamine is greater than 500 μg/mL, so the general toxicity activity was regarded weak. Therefore, if the activity persists *in vivo*, then the product could be a candidate to assess more fully for a development of a new drug for the treatment of fungal infections.

## Materials and methods

### Biological marine material

The marine sponge (Ref. EM14) *H. viscosa*(Topsent,^1888^) was collected from the Atlantic coast of El-Jadida city, Morocco (N 33 15 422, W 008° 29 722)(El-Amraoui et al. 2010). The sponge was identified by Dr. Maria-Jesús Uriz, Research Professor at the Centro de Estudios Avanzados de Blanes(CEAB), Spain. After collection, the sponge was immediately cut into small pieces, washed with sterile distilled water, then frozen at -30°C for two days and immediately freeze-dried to give a lyophilized material ready for extraction.

### General experimental procedures

Freeze-dryer was a FreeZone2.5Plus type (Labconco, USA).

TLC was carried out on precoated Macherey-Nagel Alugram silica, and spots were visualized by spraying with iodine vapor or ninhydrine reagents.

The UV spectrum was recorded on a Helios Omega spectrophotometer (Thermo Scientific, France).

The IR spectrum was obtained on a spectrometer IR-FT Paragon 1000 PC (Perkin-Elmer, USA).

The NMR spectra (^1^H, ^13^C, HSQC, HMBC, COSY and TOCSY experiments) were recorded on a Bruker 500 with a TXI cryosonde (PRISM, Rennes University, France) and a Bruker Avance 500 (CRMPO, Rennes University, France) for in-deep 2D experiments and irradiations tests.

HRESIMS data were recorded on a Micromass Zab Spec Tof (CRMPO, Rennes University, France). ESIMS data of the acetylated product was recorded on a Finnigan LCQ mass spectrometer (IFREMER, Nantes, France).

Optical rotation was measured on a Schmidt + Haensch Polartronic NH8 polarimeter.

Acetylation: 1 mg of isolated product was treated with 1 mL of acetic anhydride (Ac_2_O):pyridine (1:1 v/v) for 24 h at ambient temperature, then 40 mL of +4°C water was added and the mixture extracted with diethyl-ether (4 × 10 mL) to afford 1.2 mg of acetylated product.

### Extraction and isolation

The sample (800 g of lyophilized sponge) was homogenized with ethanol 80% (1 × 1000 mL), allowed to stand in a dark chamber for 24 h and filtered. The residue was again extracted with absolute ethanol (2 × 1000 mL). Both ethanolic extracts were combined, and then evaporated at reduced pressure until total evaporation of ethanol. The resulting aqueous suspension was completed with distilled water to 1000 mL as final volume and extracted with CH2Cl2 (3 × 500 mL). The CH2Cl2 solutions were combined, dried on anhydrous sodium sulphate (Na2SO4), filtered and concentrated at reduced pressure to give a dichloromethane extract (3 g).

This extract was fractioned by modified Kupchan method: 3 g were dissolved in 300 mL of MeOH:H_2_O (9:1 v/v) and extracted with 3 × 150 mL of hexane (A) (1.5 g). The remaining solution was adjusted with distilled water to get the proportions 6:4 v/v, then extracted with 3 × 150 mL of dichloromethane (B) (0.3 g) and then with 3 × 150 mL of ethyl acetate (C) (0.7 g). The residue was lyophilized (D) (0.5 g).

Fractions C and D were combined (1.2 g), applied on silica gel 60 (25 g) column, and eluted with successive mixtures (110 mL) of CHCl_3_: MeOH (10:0; 9:1; 8:2; 7:3; 6:4; 4:6 and 0:10 v/v) to yield nine fractions. Fractions 4, 5 and 6 were pooled (260 mg), then separated on a semi-preparative HPLC diol column (Inertsil Diol L10OH.25R, 10 × 250 mm, 10 μm) by a CH_2_CL_2_: MeOH (8.5:1.5 v/v) isocratic mixture. Purification of subfractions 2 (95 mg) by HPLC C18 column (Inertsil ODS-3, 4.6 × 250 mm, 5 μm) with isocratic MeOH:H_2_O (6.5:3.5 v/v) gave 47 mg of a pure active product.

### Microorganisms

The fungal species obtained from the Fungi Culture Collection (FCC) of the National Cultures Collection of Microorganisms of the Pasteur Institute, Paris, France, from the Collection of Institut Pasteur (CIP) and from the American Type Culture Collection (ATCC) were used as the antifungal test strains*: Candida albicans* (ATCC 10231), *Candida tropicalis* (R2 CIP 1275.81, an amphotericin B and nystatin resistant strain), *Cryptococcus neoformans* (ATCC 11576). The yeasts were maintained on the Sabouraud’s agar medium at 28°C.

### Antifungal activity

The antifungal activity was assessed *in vitro* by agar disc-diffusion test. The minimum inhibitory concentration was evaluated by the microdilution method.

#### Agar disc-diffusion test

This test uses Yeast Morphological Agar (YMA) as medium [yeast nitrogen base (Difco) 60.5 g/L; asparagin (Prolabo) 1.5 g/L; glucose (Merck) 10 g/L and agar (Merck) 20 g/L]. The suspensions of yeast were adjusted in sterile water to match the density of a 0.5 McFarland Standard. Each disk received 100 μg of sponge extract and was applied on the test media which were previously inoculated with each test strain. Plates were first kept at 4°C for at least two hours to allow the diffusion of chemicals, and then incubated at 28°C. Inhibition zones were measured after 24 h of incubation (Galeano and Martínez 2007). Standard disks of nystatin (100 μg) served as positive antifungal control. All the assays were carried out in triplicate.

#### Minimum inhibitory concentration (MIC)

The MIC_90_ (the lowest concentration causing at least 90% of growth inhibition when compared to drug-free control) of the isolated compound was measured using the method described by Rifai et al. (2005). Yeast Morphological Broth medium was used as test media. Tests were performed in 96-well round bottom sterile culture plates. The suspensions of yeast were adjusted in sterile water to match the density of a 0.5 McFarland Standard. The wells of a microdilution plate were inoculated with 180 mL of the culture medium containing a final inoculum of 0.5 - 2.5 10^3^ CFU/mL. The test drug and positive control (nystatin) previously solubilized in dimethylsulphoxide (DMSO) were serially diluted two fold in the liquid medium to give a range of concentration from 640 to 0.1 μg/mL. Twenty μL of each concentration were added to wells containing culture suspension except the growth control well. The final concentration ranged from 64 to 0.01 μg/mL. Plates were incubated at 35°C for 48 h. Fungal growth was assessed at 494 nm by measuring the optical density in each well using an enzyme immunoassay multiwell reader (Sigma diagnostic). The test was carried out in triplicate.

### Brine shrimp toxicity test

#### Hatching shrimp

Brine shrimp eggs (*Artemia salina*) are hatched in artificial seawater prepared by dissolving sea salt in distilled water (38 g/L) during 48 h incubation in a warm room (22 - 29°C). Seawater is placed in a small unequally divided tank and shrimp eggs are added to the larger compartment of the tank which is covered by aluminium foil to darken it. The illuminated compartment attracts shrimp larvae (*nauplii*) through perforations in the dam (Meyer et al. 1982).

#### Brine shrimp microwell toxicity assay

The toxicity of haliscosamine was monitored by the brine shrimp lethality test. Samples were dissolved in DMSO and diluted with sea water so that the final concentration of DMSO did not exceed 0.05%. Serial dilutions (2000, 200 and 20 μg/mL) of samples were made in wells of 96-well microplates in triplicate in 100 μL of sea water (Rahman et al. 2001). The last row was left with sea water and DMSO only served as the drug free control. 100μL of suspension of *nauplii* containing about 10 larvae were added into each well and incubated for 24 h at 22-29°C. The plates were then examined under a binocular microscope (× 12.5) and the number of dead *nauplii* in each well was counted. One hundred μL of methanol were then added and after 10 min, the total numbers of shrimp in each well was counted and recorded. Lethality concentration fifties (LC_50_ values) for each assay were calculated by taking the average of the three experiments using a Finney Probit analysis program on an IBM computer.
